# Localized Amyloidosis of the Upper Aerodigestive Tract: Complex Analysis of the Cellular Infiltrate and the Amyloid Mass

**DOI:** 10.1155/2019/6165140

**Published:** 2019-08-19

**Authors:** Lilla Turiak, Bálint Kaszás, Krisztián Katona, Ágnes Lacza, László Márk, Károly Vékey, László Drahos, Tamás Tornóczky

**Affiliations:** ^1^Institute of Organic Chemistry, Research Centre for Natural Sciences, Hungarian Academy of Sciences, Budapest, Hungary; ^2^Department of Pathology, Oral Pathology Unit, Medical School and Clinical Center, Pécs University, Hungary; ^3^Department of Dentistry, Oral and Maxillofacial Surgery, Medical School and Clinical Center, Pécs University, Hungary; ^4^Department of Biochemistry, Medical School, Pécs University, Hungary

## Abstract

**Objectives:**

The aim of this study was to analyse the composition of amyloid mass and the plasmacytic infiltrate of localized amyloidosis of the upper aerodigestive tract.

**Methods:**

Biopsy materials were studied by light microscopy, immunohistochemistry (IHC), and mRNA in situ hybridization (mRNA-ISH). The amyloid mass was also analysed with high-performance liquid chromatography mass spectrometry- (HPLC-MS-) based proteomics.

**Results:**

Nodular and diffuse forms of amyloid deposition were detected. IHC analysis revealed *λ*-light chain (LC) in two cases, *κ*-LC in one case. The remaining two were positive with both. Proteins, well known from other amyloidoses like amyloid A (AA), prealbumin/transthyretin (PA), apolipoprotein A-I (ApoAI), and amyloid P component (APC), and also keratin were found with variable intensities in the cases. HPLC-MS revealed dozens of proteins with both LCs in all the lesions but sometimes with surprisingly small intensities. mRNA-ISH analysis revealed identical *λ* and *κ* dominance and only one normal *κ*/*λ* cell ratio.

**Conclusion:**

Cellular infiltrate and protein components in the amyloid showed congruent results in all but one case. The only exception with normal cell ratio and *λ*-dominant amyloid could be originated from the different protein-secreting activity of plasma cell clones. HPLC-MS analysis explored both LCs in all the amyloid in variable amount, but other proteins with much higher intensities like keratins, apolipoprotein A-IV (ApoAIV), were also detected. Proteins like AA, PA, ApoAI, and APC, previously known about amyloid-forming capability, also appeared. This indicates that localized amyloid in the upper aerodigestive tract is not a homogenous immunoglobulin mass but a mixture of proteins. The sometimes very low light chain intensities might also suggest that not all the localized amyloidosis cases of the upper aerodigestive tract are of convincingly AL type, and the analysis of the cellular infiltrate might indicate that not all are monoclonal.

## 1. Introduction

Localized amyloidosis is a rare disease with deposition of amyloid material in a restricted area of an anatomical region, which results in a tissue mass. This “mass effect” may mimic tumour growth explaining the other name of the lesion: amyloid tumour. The type of the protein locally deposited is variable and related to the anatomical site: A*β* (*β* protein precursor) in the central nervous system, AL (light chain) in the upper aerodigestive tract, AIAPP (islet amyloid polypeptide) in the pancreas islets, ASem1 (semenogelin) in the seminal vesicles, AGal7 (galectin 7) and AIns (insulin) in the skin, APro (prolactin) in the pituitary gland, and AANF (atrial natriuretic factor) in the atrial myocardium just to mention some of them [1]. The AL type is by far the most frequent localized amyloidosis [2]. Despite the relatively large number of case reports on localized AL amyloidosis, the majority of which mainly summarize the clinicopathological characteristics of the disease; little is known about the pathogenesis and the composition of the amyloid material. AL amyloid is supposed to be the product of locally accumulated plasma cells, which are regarded to be of monoclonal origin. In addition, very frequently just few plasma cells could be identified in the biopsy material which led to the “suicide neoplasm” theory [3]. Moreover, amino acid sequence analysis of amyloid light chain proved the monoclonal nature of the protein (either *κ* or *λ*) in those cases examined [4, 5]. If it was abundant, the cellular component in the lesions was also analysed and frequently found to be monoclonal or obviously neoplastic [6–11]. Polyclonal nature of the infiltrating plasma cells was also found in some cases which seemingly contradicts the neoplastic nature of the lesion [8]. Few years ago, as a new method, mass spectrometry (MS) was introduced in the amyloid protein analysis of the systemic diseases, and in a recent study, MS was combined with the standard immunohistochemistry (IHC) to identify the nature of the protein [12, 13]. 100% concordance was found between the two methods [14]. Further combination of MS with laser capture microscopy made the diagnostics of amyloidosis even more effective [15]. IHC analysis of some of the localized amyloidosis cases of our departmental archive indicated the presence of both light chains in the amyloid material, which raised the possibility that the AL amyloid deposits could also be polyclonal products. The composition of the amyloid material and the cellular component in localized disease are not extensively studied; therefore, we decided to extend the immunohistochemistry and complete the study with mRNA in situ hybridization and proteomics using nano-HPLC-MS to determine the clonality of the cellular infiltrate and to further analyse the amyloid material in our cases.

## 2. Materials and Methods

### 2.1. Patients and Biopsy Samples

Five patients (four male and one female) with localized amyloidosis of the upper aerodigestive tract were collected from the archive of the pathology department. Three lesions appeared in the larynx (vocal cord, vocal fold, and supraglottic region) and two (one each) in the epipharynx and the oral cavity (palate). The age range was 31-62 years, and in all the cases, the main clinical symptom was a mass-like lesion in the affected region suggesting malignancy (Table 1). Despite this, none of the patients developed systemic or malignant disease according to the clinical data explored.

### 2.2. Histopathological Evaluation

Excision biopsies from the five cases were analysed. After the standard preanalytical processes (fixation in 6% buffered formalin, dehydration, and paraffin embedding of the tissue samples), 4 *μ*m thick sections were cut and standard haematoxylin-eosin (HE) stainings were prepared for diagnostic purposes. The Congo red staining was performed with and without KMnO_4_-trypsin pretreatment according to Romhanyi's method, and the slides were studied in polarized light [16]. Kossa reaction was performed when calcification of the amyloid material was seen. Parallel sections were used for proteomic analysis (see below).

### 2.3. Immunohistochemistry

*κ* and *λ* immunohistochemical stainings (monoclonal antibodies, Thermo Scientific, L1C1 and HP6054 clones in dilutions of 1 : 4000 and 1 : 2000, respectively, at 60 minutes in room temperature) were used to identify the type of light chains in the amyloid material and in the plasma cells. DAKO Autostainer Link 48 platform was used for the reactions, and the Envision Flex TRS high pH (DAKO) antigen retrieval solution was selected for antigen retrieval. The pretreatment was performed at pH 9 for 20 minutes. Histols MR and DAB Histols MR (30 and 10 minutes, respectively, at room temperature) were used as developing system. CD38 mouse monoclonal antibody (clone SPC32, Leica Biosystems) was used to identify plasma cells. The staining was run at the Leica Bond-Max platform for 15 minutes at 1 : 150 antibody dilution, and the developing system was Bond Polymer Refine Detection Kit (Leica Biosystems) with DAB chromogen. The pretreatment was performed with Bond Epitope Retrieval Solution 1 (Leica Biosystems) at pH 6 for 20 minutes. The same platform, pretreatment, and developing system were used for the primary antibodies against amyloid A (AA, mouse monoclonal anti-human amyloid A, mc1, DAKO, 1 : 200, pH 9, 20 minutes), prealbumin/transthyretin (PA, polyclonal rabbit anti-human prealbumin, DAKO, 1 : 500, pH 6, 20 minutes), apolipoprotein A-I (ApoAI, mouse monoclonal ApoAI, clone 6001, Thermo Fisher, 1 : 1000, pH 6, 20 minutes), amyloid P component (APC, rabbit polyclonal serum amyloid P, Thermo Fisher, 1 : 200, pH 9, 20 minutes), and pan-cytokeratin (mouse monoclonal AE1-AE-3, DAKO, 1 : 300, pH 9, 20 minutes).

### 2.4. mRNA In Situ Hybridization

The automated Leica Bond-Max system was used in this assay as well. 4 *μ*m thick FFPE sections were deparaffinised at 60°C for 30 minutes in Bond Dewax Solution and digested with proteinase-K (Enzyme Pretreatment kit) at 37°C for 15 minutes. *κ* and also *λ* mRNA in situ hybridizations (mRNA-ISH) were performed on all samples using the Bond Ready-to-Use ISH kit at 37°C for 120 minutes. After a short period of endogenous peroxidase blocking for 5 minutes, incubation with Anti-Fluorescein Antibody (15 minutes, room temperature) and Bond Polymer Refine Detection system DAB were used for detecting the signals (all from Leica Biosystems).

### 2.5. Digitization of mRNA-ISH Slides and Image Analysis of Cellular Infiltrate

*κ* and *λ* mRNA-ISH stained slide pairs cut from the same paraffin blocks of the cases were digitized (Pannoramic MIDI, 3DHistech) consecutively, and the image analysis was performed on the virtual slides. The aim of the analysis was to determine the ratio of the *κ*- and *λ*-positive cells. Two to three equivalent annotations were made in the slide pairs by CaseViewer (3DHistech) in the cell-rich areas. PatternQuant module (3DHistech) was used to select the corresponding tissue areas and to exclude the amyloid material and the cell-free fields. Plasma cells positive for *κ* or *λ* mRNA were counted by CISH-RNAQuant module (3DHistech) independently of their staining intensity in all the annotations after visual adjustment. The ratio of *κ*/*λ*-positive plasma cells was calculated in percent (see Table 1). Normal *κ*/*λ* ratio was determined as 2/1 (67%/33%) according to a former publication [17].

### 2.6. Nano-HPLC-MS(MS) and Proteomic Analysis

Unstained formalin-fixed paraffin-embedded (FFPE) slides were dewaxed, and antigen retrieval was performed as previously described [18]. Tissue sections were dried, and regions corresponding to amyloid mass in the HE-stained slides were scraped off using a needle and placed in 50 *μ*L lysis buffer (4% SDS in 100 mM Tris pH 7.6). Proteins were precipitated using ethanol and digested in solution [19]. The peptides were desalted using Pierce C18 spin columns (Thermo Scientific, Waltham, MA, USA). Discovery proteomics was performed using an Ultimate 3000 nanoRSLC system (Dionex, Waltham, MA, USA) coupled to a high-resolution Bruker Maxis II ETD Q-TOF mass spectrometer (Bruker Daltonics, Bremen, Germany) via a CaptiveSpray NanoBooster insource [20]. Samples were dissolved in 20 *μ*L of 2% AcN, 0.1% FA out of which 6 *μ*L was analysed. Protein identification was performed using the Mascot server v.2.5 (Matrix Science, London, UK). Samples were matched against the Swissprot human database (2015_08) applying the following search parameters: trypsin enzyme, maximum 2 missed cleavages, 7 ppm peptide tolerance, 0.05 Da fragment mass tolerance, and carbamidomethylation as fixed modification and carbamyl (K, N-term), deamidation (N, Q), and oxidation as variable modifications. Proteins with a minimum of two identified unique peptides were accepted. Label-free quantification was performed using MaxQuant software version 1.5.3.30 [21].

## 3. Results

### 3.1. Histopathological Analysis

**Figure 1:**
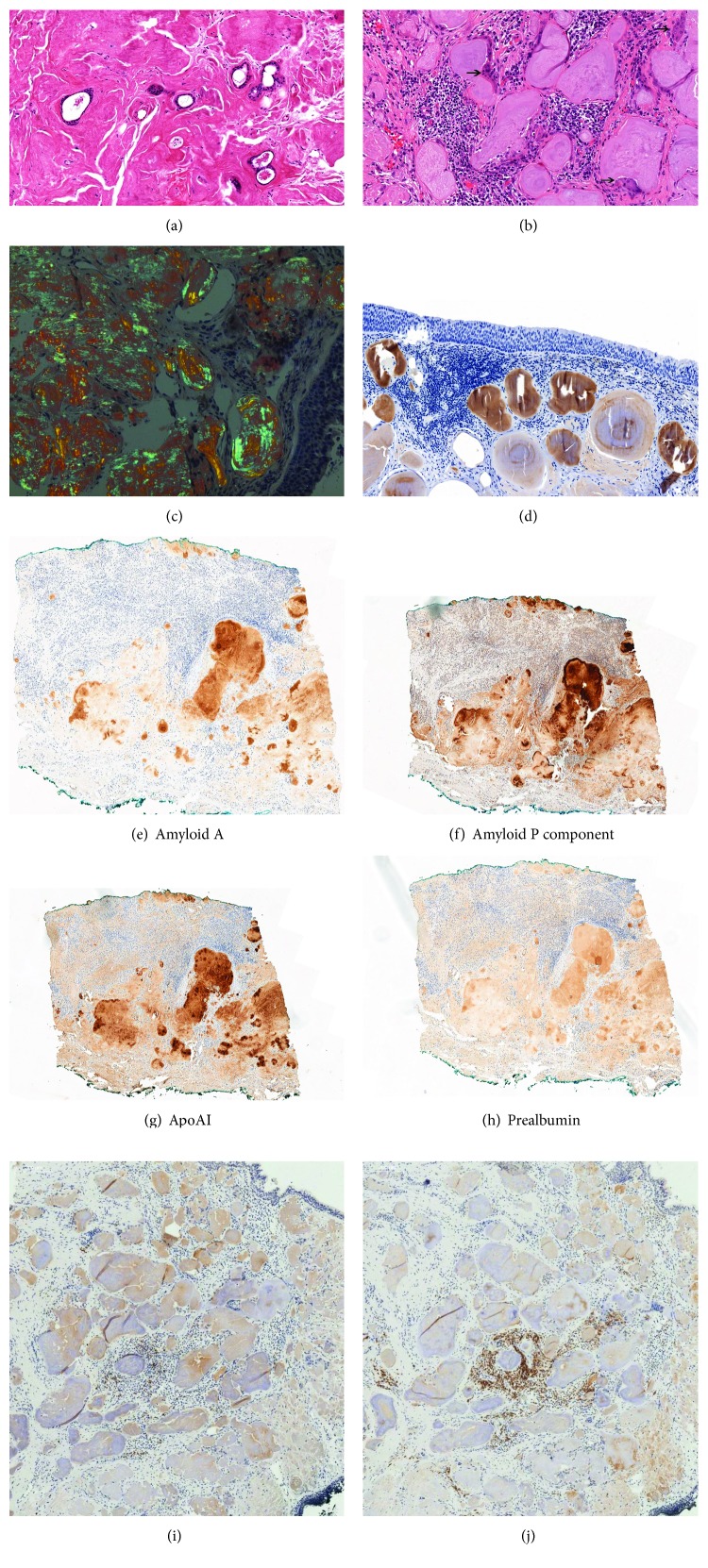
(a) HE staining, low power magnification. The photograph demonstrates the diffuse, infiltrating, type of amyloid deposition in biopsy material of patient 2. See how the eosinophilic amyloid material surrounds and embeds the mucus ducts. (b) HE staining. This midpower detail (patient 5) represents another form, the concentrically laminated nodular appearance of amyloid deposition. Note the occasional multinucleated giant cells (arrows) surrounding the nodules and the lymphoplasmacytic infiltrate in between. (c) Congo red staining, polarized light, high-power magnification. The biopsy specimen of patient 1 shows the laminated structure of the Congo red-positive amyloid nodules with apple-green birefringence. (d) *λ* immunohistochemical staining, midpower photograph, patient 1. Note the respiratory epithelium on the top and the immunopositive nodules showing different staining intensity and laminated structure. (e–h) Immunopositivity for amyloid A, P component, ApoAI, and prealbumin, respectively, in patient 4. Note the variable staining intensity in the amyloid material. (i, j) *κ* and *λ* mRNA-ISH on consecutive slides of patient 5. See the *λ*-dominant, centrally placed plasmacytic population in (j).

In all examined cases, the largest proportion of the overall tissue volume was formed by the amyloid material. Structurally, the deposition appeared in the form of a diffuse mass infiltrating and embedding the surrounding tissue elements like glands and collagen fibres, or it formed concentrically laminated nodules sitting side by side (Table 1). In cases 1, 4, and 5, the two patterns were mixed with each other (Figures 1(a) and 1(b)). All the cases showed moderate to strong Congo red staining and apple-green birefringence in polarized light even after KMnO_4_-trypsin pretreatment (Figure 1(c)). In some of the cases (patients 1 and 4), giant cell reaction around and focal calcification within the amyloid material were also seen (Figure 1(b)). Although a variable number of white blood cells (plasma cells, lymphocytes) were detected in the samples by the HE and CD38 staining, the amount of the cellular infiltrate was only a fraction of the amyloid material. In some cases, the cellular component was very scarce and the *κ* and *λ* immunohistochemical analyses failed to identify the clonality of the population. Unambiguous result was found only in case 4 which showed diffuse strong *κ* positivity in the plasma cell population, and just a few reactive *λ*-positive cells were detected. In two of the cases (patients 1 and 5), both *κ* and *λ* antibodies were positive in the amyloid deposits; in the other two cases (patients 2 and 3), only the *λ* light chain could be detected while in the remaining one (patient 4) only *κ* was convincingly positive in the deposits (Table 1). The intensity of the immunostaining was variable, and the pattern was similarly laminated (stronger and weaker layers alternating with each other) in the nodules as previously seen in HE (Figure 1(d)). The four proteins (AA, PA, ApoAI, and APC) partly known because of their amyloidogenic potential were frequently found in the amyloid mass of the patients 1, 2, 4, and 5 (Table 2). The intensity of the reactions was also variable even in the same sample frequently highlighting the structure of the nodules (Figures 1(e)–1(h)). APC showed a strong peripheral staining pattern (Figure 1(f)). Weak immunoreaction of pan-cytokeratin was detected in cases 1 and 5, but no reaction was seen in case 4 (Table 2). Due to the small size of the original biopsy, no tissue remained in cases 2 and 3 in order to examine the protein expressions above.

### 3.2. mRNA In Situ Hybridization

Analysis of the identical fields of mRNA-ISH specimens revealed *κ*- or *λ*-dominant plasma cell populations in four cases (Table 1). In patients 1, 3, and 5, *λ*-dominant cell infiltrate was seen with 19% vs. 81%, 5% vs. 95%, and 16% vs. 84% *κ*/*λ* ratios, respectively. In patient 4, *κ*-dominant population was observed: the *κ*/*λ* ratio was 86% vs. 14%. In patient 2, this ratio was 67% vs. 33%, which is similar to the normal human reactive lymphoid population with its 2 : 1 ratio [17]. Dominance of one of the plasma cell types was unequivocal in all but one case (patient 2), which qualifies them as rather clonal plasma cell infiltrate (Figures 1(i) and 1(j)). The cellular infiltrate was, however, intensive enough in only one case to raise the possibility of a local plasma cell neoplasm (patient 4). IgH-PCR analysis was also performed, but the results were inconclusive; therefore, the data were not listed here.

### 3.3. Nano-HPLC-MS(MS) Analysis of Amyloid Mass

**Figure 2:**
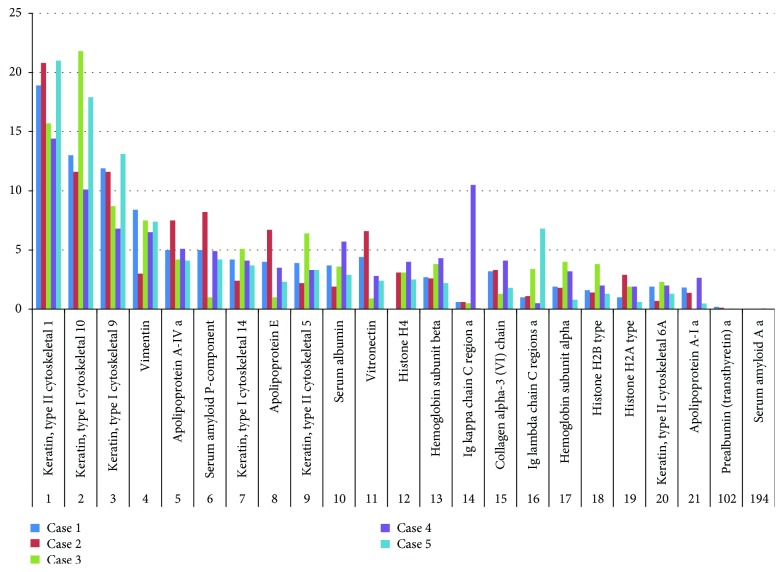
List and relative distribution of the top 21 and 2 low abundance proteins observed by nano-HPLC-MS(MS) and given in percent in the five cases; normalized to the top 21 proteins. Known amyloidogenic proteins, like ApoAIV (5), LC *κ* and *λ* (14 and 15), ApoAI (21), PA (102), and AA (194) partly examined by immunohistochemistry, were also present in the amyloid material in different concentrations. Note that not just LCs but also ApoAIV and ApoAI with higher intensity and PA and AA with much less intensity also participated in the formation of the lesion. The numbers at the bottom of the columns indicate the mean frequency of the particular protein.

The amyloid mass region of the dewaxed tissue slides were scraped off and used for proteomic analysis, using high-resolution nano-HPLC-MS/MS setup, as described above. Amyloid deposits showed approximately 500 proteins (detected with at least 2 unique peptides altogether in the five samples). Among these, various IgG components including *κ* and *λ* light chains were detected [14].  Figure 2 summarizes the percent distribution for the 21 most abundant and two low abundance proteins in the five cases studied. These include ApoAIV, APC, apolipoprotein E (ApoE), and vitronectin, which were previously proved to participate in the formation of amyloid [1, 3]. Other amyloidogenic proteins, like AA and PA, were also detected in low abundance, similar to the IHC results (Figure 2). Surprisingly, both the *κ* and *λ* light chains were present in each sample. Eight unique peptides corresponding to the Ig kappa chain constant region (IGKC_HUMAN) were detected. The Ig lambda chain constant region was detected also by 8 peptides. In the samples, peptides corresponding to the variable regions of the Ig kappa and Ig lambda chains were also found in low abundance. To determine total immunoglobulin content and the ratio of the *κ* and *λ* light chains in the samples, label-free quantitation was performed using the MaxQuant software, utilizing peptides in the constant regions. The ratio of the *κ*/(*κ*+*λ*) light chains in the studied samples is shown in Table 3. Values close to 1 indicate *κ* dominance, while values close to 0 indicate *λ* dominance. In patients 3 and 5, the values were 0.12 and 0.02, respectively, suggesting *λ* dominance. This ratio was 0.94 in patient 4 indicating *κ* dominance. The remaining two cases (patients 1 and 2) had similar *κ*/(*κ*+*λ*) ratios (0.26 and 0.29) suggesting lambda dominance. Based on the label-free quantitative results, the total immunoglobulin amount of the amyloid mass varied between 2 and 9% in the cases analysed, while the total kappa and lambda chain contents were between 0.1-7% and 0.4-5%, respectively. Individual results of the examined cases are summarized in Table 3. Changes in the amount of *κ* and *λ* light chains were determined relative to the total amount of all the proteins detected in the amyloid mass. We recalculated the values incorporating the variable regions of the kappa and lambda chains as well. It resulted in minor changes only and did not have an impact on the results.

## 4. Discussion

The neoplasm-mimicking clinical appearance and the most frequent anatomical sites of localized amyloidosis are well-known characteristics mainly from case studies [2, 22–26]. Our series of localized amyloidosis showed the previously published features. Three of the five occurred in the larynx and the other two in the epipharynx and oral cavity (palatum), respectively. Histological examination of the biopsies explored common changes. On HE-stained slides, the amyloid material appeared either in the form of a diffuse eosinophilic infiltrate embedding the normal tissue structures or combined with concentrically laminated nodular form of amyloid deposition (Figures 1(a) and 1(b)). This is probably due to the different organization of the protein molecules in the amyloid, but the details are not understood; these two different patterns of deposition are not widely examined and not frequently reported features. None of the above patterns linked to any specific light chains or other amyloidogenic proteins by immunohistochemistry, all the proteins appeared in both forms of deposition. Besides the occasional calcification, foreign body type of giant cells was also visible mainly around the nodular deposits. These cells are thought to participate in amyloid material processing [3, 27]. Both forms of depositions were Congo red-positive and showed birefringence under crossed polars with apple-green polarization colour even after the KMnO_4_-trypsin pretreatment.

It is generally accepted that the cellular infiltrate in the localized amyloidosis is monoclonal [8–11]. All the lesions except case 4 in this study were hypocellular. Probably, this was the reason of the failure of IgH-PCR analysis in characterizing the clonality of the cellular infiltrate. The *κ* and *λ* immunohistochemistry also did not produce reliable results since the staining intensity in the cellular infiltrate and the number of the cells were generally low even on the CD38-stained slides (except case 4). Based on the intensity and specificity of staining on the consecutive slides, reliable results were obtained with the *κ* and *λ* mRNA-ISH. Image analysis revealed *κ* or *λ* dominance in all but one sample suggesting the clonal origin of the plasmacytic population. Biopsy material from patient 2, however, showed 2 : 1 *κ*/*λ* ratio which is in the normal range indicating the polyclonal nature of the cellular infiltrate in this case. In patients 1, 3, and 5, *λ* dominance was seen at a ratio of 4x, 19x, and 5x, respectively (Table 1), while in patient 4 6x *κ* dominance was calculated by the analysis. Despite the numerous literature data on the monoclonal nature of the lesion, some cases—similar to our patient 2—are clearly polyclonal. Moreover, many monoclonal examples, three cases from localized amyloidosis and a nodular pulmonary amyloidosis series published earlier, revealed polyclonal cellular infiltrate, which raises the possibility that some lesions are not neoplastic [8, 28]. This seemingly contradicts the suicide neoplasm theory [3].

The composition of amyloid material in localized amyloidosis is not extensively studied and not a published area of histopathology. HPLC-MS analysis of the amyloid material revealed *κ* dominance in patient 4 and *λ* dominance in patients 1, 2, 3, and 5, respectively. All but one of these proteomic results are in accordance with the dominance of the plasma cell population determined by the mRNA-ISH; therefore, in these cases a localized clonal plasma cell disease might be suggested in the background. Despite the frequently obvious *κ* or *λ* dominance, no local or systemic neoplasm could be identified in the clinical history of the patients. This may draw the attention that a *κ*- or *λ*-dominant population is not necessarily a neoplasm at the same time. We should emphasize that besides the dominant form, the other light chain was also present in the amyloid material, probably due to the insudation from the blood plasma or true local light chain production by the other plasma cell population. In the biopsy sample of patient 2, *λ* dominance was measured in the amyloid by HPLC-MS, which is in contrast to the polyclonal nature of the plasmacytic infiltrate defined by the mRNA-ISH. This is interesting and obscure but not unique since a similar case was published by Grogg et al. in a nodular pulmonary amyloidosis series (case 9), where *κ* light and *γ* Ig heavy chains were identified by mass spectrometry beside the mixed plasma cell population [28]. The contrast between the polyclonal plasmacytic population and *λ* dominance in the proteomics in our case 2 may reflect on a different protein synthesis activity of the *κ*- and *λ*-positive plasmacytic clones, or it might be explained by the more intensive amyloidogenic nature of the *λ* light chains. The immunohistochemical finding of the amyloid was congruent with the results of the HPLC-MS analysis in patients 3 and 4 and also identified the other light chain (*κ*) in the otherwise *λ*-dominant amyloid material of patients 1 and 5. In these two later cases, HPLC-MS also detected the *κ* chain. Both light chains in approximately equal amounts were identified by the two methods in the amyloid material of a lesion on the palate of a patient [29].

HPLC-MS also showed that similar to the systemic forms, the amyloid material in the localized amyloidosis of the upper aerodigestive tract is by far not a homogenous immunoglobulin mass but rather a mixture of structural, cytoskeletal, regulatory, or serum proteins with different intensities in which the light chains sometimes produce just a smaller part of the mass (Figure 2). Cytokeratins, vimentin, collagen, haemoglobin, apolipoproteins, and albumin may all be derived partly from the serum or from the surrounding tissue structures destroyed by the accumulating amyloid mass. Moreover, both IHC and HPLC-MS revealed three well-known, potentially amyloidogenic proteins (AA, PA, and ApoAI) in the lesions, but their primary amyloid-forming nature in the localized amyloidosis of the upper aerodigestive tract has not been proven yet.

Besides these, other proteins like ApoAIV, ApoE, APC, and vitronectin, the well-known components of the so called “universal amyloid proteome” in systemic amyloidosis, were all identified in our localized amyloidosis cases with much higher intensities than the former three [30]. ApoE, ApoAI, ApoAIV, collagen, and vitronectin were also identified by mass spectrometry in a nodular pulmonary amyloidosis series published recently [28]. These facts may emphasize the partly similar composition of the amyloid material in the localized and systemic diseases; however, the true fibril-forming components could be identified only by high-resolution microscopic methods.

Although IHC indicated keratin just in two of the three cases, HPLC-MS identified keratin 1, 10, 9, 14, and 5 in the amyloid mixture of the samples. Sometimes the keratins appeared with the highest intensities in the cases analysed; however, we should emphasize that HPLC-MS or IHC could indicate only the presence and intensity of these proteins in the mass and not their primary amyloid-forming nature.

Similar to the nodular pulmonary amyloidosis series cited above, the intensity data of ApoAIV were also relatively high in all the samples examined in this study which might indicate its initiator role in the localized amyloidogenesis [28]. Several years ago, a case of systemic amyloidosis of two separate proteins (transthyretin and ApoAIV) with distinct deposition patterns was described, and recently, in our university, ApoAIV type of systemic amyloidosis was discovered in a PACAP-KO mice strain, which all may indicate the primary amyloidogenic potential of the ApoAIV protein [31, 32].

HPLC-MS analysis, histology, and mRNA-ISH were proved to be a powerful combination in the characterization of these complex lesions. The frequent low intensity of the immunoglobulins and high intensity of other proteins might raise the latter's primary role in the genesis of localized amyloidosis in the upper aerodigestive tract.

## Figures and Tables

**Table 1:** Characteristics of localized amyloidosis cases of the upper aerodigestive tract. Congo staining was performed with and without KMnO_4_-trypsin pretreatment, and the slides were analysed also in polarized light. The type of cellular infiltrate and the structural and immunohistochemical characteristics of amyloid deposition were also analysed. Patient: m: male; f: female; Congo: staining with/without KMnO_4_-trypsin pretreatment; IHC: *κ* and *λ* immunohistochemical staining feature of the accumulated amyloid; mRNA-ISH: messenger RNA in situ hybridization of plasma cells in the cellular infiltrate.

Patient		Anatomic site	Macroscopy	Congo	Amyloid	Microscopy	IHC	mRNA-ISH
1	61, m	Larynx (vocal+ventricular fold)	Verrucous mass	+/+	Diffuse+laminated concentric	ly+plasma cell	*κ*+, *λ*+	*κ*/*λ*: 19%/81% (1 : 4)
2	44, m	Epipharynx	Cyst	+/+	Diffuse	ly+plasma cell	*κ*−, *λ*+	*κ*/*λ*: 67%/33% (2 : 1)
3	51, f	Larynx (vocal fold+supraglottis)	Mass	+/+	Diffuse	ly+plasma cell	*κ*−, *λ*+	*κ*/*λ*: 5%/95% (1 : 19)
4	62, m	Oral cavity (palate)	Mass	+/+	Diffuse+laminated concentric	ly+plasma cell	*κ*+, *λ*−	*κ*/*λ*: 86%/14% (6 : 1)
5	31, m	Larynx (ventricular fold, right)	Mass	+/+	Diffuse+laminated concentric	ly+plasma cell	*κ*+, *λ*+	*κ*/*λ*: 16%/84% (1 : 5)

**Table 2:** Immunohistochemical reaction of the four amyloidogenic proteins and cytokeratin examined on the amyloid material of the five cases. The intensity of the reactions was variable highlighting the concentric laminar structure of the nodular mass. NA^∗^: data are not available. Due to the lack of amyloid or tissue material, the reactions were not evaluated. CK: pan-cytokeratin AE1-AE3, only weak positivity^∗∗^.

Patient	Prealbumin	ApoAI	Amyloid A	Amyloid P component	CK^∗∗^
1	+	+	+	+	+
2	+	+	−	−	NA^∗^
3	NA^∗^	NA^∗^	NA^∗^	NA^∗^	NA^∗^
4	+	+	+	+	−
5	+	+	+	+	+

**Table 3:** Summary of the nano-HPLC-MS(MS) results. Percents of total immunoglobulin, Ig *κ* chain constant region, Ig *λ* chain constant region, and *κ*/(*κ*+*λ*) ratio were determined by label-free quantitation and normalized to total amyloid mass. The *κ*/(*κ*+*λ*) ratio of the cases was determined by nano-HPLC-MS(MS) using label-free quantitation. Due to the presence of low abundance Ig components, data were normalized to total amyloid mass, resulting in small numerical differences compared to Figure 2.

	Age/gender	Anatomic site	Nano-HPLC-MS(MS) total immunoglobulin (%)	Nano-HPLC-MS(MS) *κ* chain (%)	Nano-HPLC-MS(MS) *λ* chain (%)	Nano-HPLC-MS(MS) *κ*/(*κ*+*λ*)
1	61, male	Larynx (vocal+ventricular fold)	2.82%	0.41%	1.16%	0.26
2	44, male	Epipharynx	1.99%	0.39%	0.95%	0.29
3	51, female	Larynx (vocal fold+supraglottis)	3.34%	0.40%	2.84%	0.12
4.	62, male	Oral cavity (palate)	9.28%	6.89%	0.40%	0.94
5.	31, male	Larynx (ventricular fold, right)	6.12%	0.10%	4.63%	0.02

## Data Availability

The data used to support the findings of this study are included within the article.

## Abbreviations

IHC: Immunohistochemistry
mRNA-ISH: Messenger RNA in situ hybridization
HPLC-MS: High-performance liquid chromatography and mass spectrometry
HE: Haematoxylin-eosin
DAB: 3,3′-Diaminobenzidine
FFPE: Formalin-fixed paraffin-embedded
IgH-PCR: Immunoglobulin heavy chain gene polymerase chain reaction
AL: Amyloid light chain
AA: Amyloid A
PA: Prealbumin/transthyretin
ApoAI: Apolipoprotein A-I
ApoAIV: Apolipoprotein A-IV
APC: Amyloid P component
IgG: Immunoglobulin G.
